# The Effects of Carbon Dioxide Removal on the Carbon Cycle

**DOI:** 10.1007/s40641-018-0104-3

**Published:** 2018-06-14

**Authors:** David P. Keller, Andrew Lenton, Emma W. Littleton, Andreas Oschlies, Vivian Scott, Naomi E. Vaughan

**Affiliations:** 1 GEOMAR Helmholtz Centre for Ocean Research Kiel, Düsternbrooker Weg 20, 24105 Kiel, Germany; 2 CSIRO Oceans and Atmosphere, Hobart, Australia; 3 Antarctic Climate and Ecosystems Cooperative Research Centre, Hobart, Australia; 4 College of Life and Environmental Sciences, University of Exeter, Exeter, UK; 5 School of GeoSciences, University of Edinburgh, Edinburgh, UK; 6 Tyndall Centre for Climate Change Research, School of Environmental Sciences, University of East Anglia, Norwich, UK

**Keywords:** Climate change, Carbon dioxide removal (CDR), Mitigation, Carbon cycle, Negative emissions, Carbon cycle feedbacks, Climate feedbacks

## Abstract

Increasing atmospheric CO_2_ is having detrimental effects on the Earth system. Societies have recognized that anthropogenic CO_2_ release must be rapidly reduced to avoid potentially catastrophic impacts. Achieving this via emissions reductions alone will be very difficult. Carbon dioxide removal (CDR) has been suggested to complement and compensate for insufficient emissions reductions, through increasing natural carbon sinks, engineering new carbon sinks, or combining natural uptake with engineered storage. Here, we review the carbon cycle responses to different CDR approaches and highlight the often-overlooked interaction and feedbacks between carbon reservoirs that ultimately determines CDR efficacy. We also identify future research that will be needed if CDR is to play a role in climate change mitigation, these include coordinated studies to better understand (i) the underlying mechanisms of each method, (ii) how they could be explicitly simulated, (iii) how reversible changes in the climate and carbon cycle are, and (iv) how to evaluate and monitor CDR.

## Introduction

**Fig. 1:**
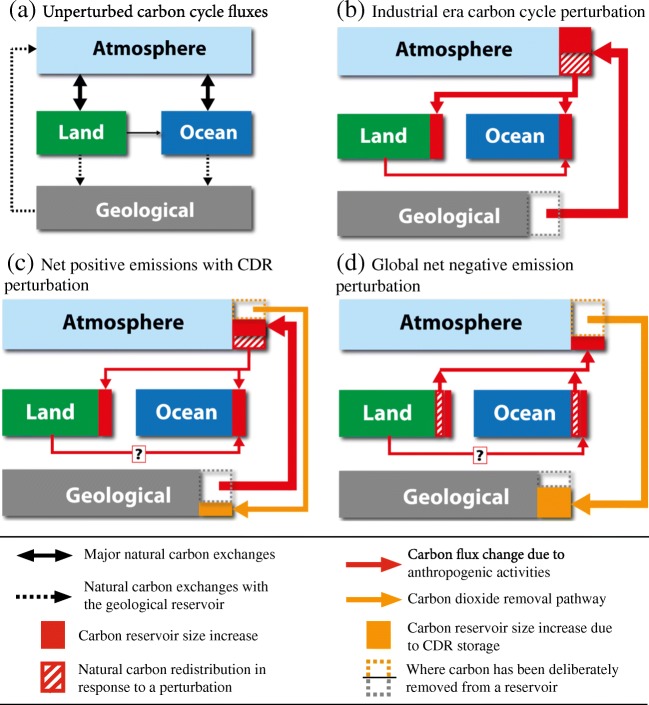
Schematic representation of the main carbon flows among atmospheric, land, ocean, and geological reservoirs for (**a**) the Earth before significant anthropogenic impacts; and how carbon flows have or may have changed due to anthropogenic activities such as (**b**) industrial era fossil fuel combustion, (**c**) when carbon dioxide removal (CDR) begins, but net CO2 emissions are positive, and (**d**) when CO_2_ is removed from the atmosphere, i.e., “net negative emissions.” Note that when net emissions are negative as in (**d**), it is still possible to have some emissions, but these are not depicted here. Carbon exchanges depicted in (**a**; black and dashed lines) also occur in **b**, **c**, and **d**. The question mark in the land to ocean carbon flux perturbation in **c** and **d** indicates that it is unknown how or if this carbon cycle perturbation will be affected by CDR. Adapted from [1••, 71•]

The Earth’s climate and the carbon cycle are inherently linked. Carbon cycle processes determine the flow of carbon between reservoirs, (Fig. 1a). In the atmosphere, the carbon-containing gases carbon dioxide (CO_2_) and methane (CH_4_), along with water vapor, are the major greenhouse gases (GHGs). These GHGs absorb a proportion of the Earth’s emitted long-wavelength radiation, thereby trapping heat. CO_2_ in particular is a very long-lived greenhouse gas, whose atmospheric concentration has been rising at unprecedented rates due to continued intensive fossil fuel use, land use change, and cement production.

Concentrations of atmospheric CO_2_ have now exceeded 400 pm, and are currently at levels not seen in at least the last 800,000 years [3]. This has led to detectable warming of the Earth, and changes in the global carbon cycle [3].

As atmospheric CO_2_ levels continue to rise the likelihood of “severe, pervasive, and irreversible” impacts increases [4]. This was recognized by the United Nations Framework Convention on Climate Change, who facilitated the Paris Agreement on climate change [5] in which countries pledged Nationally Determined Contributions (NDCs) to deliver emissions reductions. However, the emissions reductions resulting from current NDCs appear to be insufficient to limit warming to “well below 2° C above pre-industrial,” the goal of the Paris Agreement [6]. Consequently, it is increasingly likely that some form of carbon dioxide removal (CDR) will be needed [7–10] to reach this goal.

CDR encompasses a range of methods aimed at reducing atmospheric CO_2_ levels either by directly extracting CO_2_ from the atmosphere or by deliberately enhancing land and ocean carbon sinks to increase removal of CO_2_ from the atmosphere. While there is clearly an overlap between CDR and mitigation actions, here, we focus on widely discussed methods that propose the deliberate uptake of atmospheric CO_2_, not the reduction of input of CO_2_ (or avoided emissions). Furthermore, we focus on CDR at a sufficiently large scale to have global impact on the carbon flows in and out of the atmosphere (see Table 1).

**Table 1:** Description of proposed carbon dioxide removal (CDR) methods where enough literature (> 2 publications that investigate carbon cycle responses) exists to begin understanding how the carbon cycle may respond to large-scale (e.g., > 1Pg C) deployment

Method	General description	References
Direct air capture of CO_2_ with storage	Technology that chemically or electro-chemically removes CO_2_ from air and concentrates it for storage	[11, 13, 14, 15••, 16–21, 126]
Bioenergy with carbon capture and storage	Grow terrestrial vegetation* and use the biomass to create biofuels that can be burned in conjunction with carbon capture and storage technology	[1••, 60, 127, 128, 101, 102, 65, 26–35, 56]
Afforestation/reforestation	Plant or restore forests to increase CO_2_ uptake (via primary production) and storage in biomass and soils	[1••, 22, 36, 37•, 42–53, 71•, 79, 70, 117]
Soil and land carbon management	Employ management practices, such as no-till agriculture, irrigation, cover crops, compost amendments, wetland restoration, and fire management, to increase C retention and storage in agricultural soils or managed natural lands	[57–59, 61–65, 67, 71•, 120]
Biochar	Pyrolyze terrestrial biomass* to form biochar and add it to soils where the C can remain sequestered (biochar is recalcitrant); biochar amendments may also enhance vegetation productivity and soil carbon storage	[23–25, 55, 61–65, 71•]
Enhanced weathering on land	Spread alkaline minerals on land to chemically remove CO_2_ from the atmosphere in reactions that form ions, which are eventually transported to the ocean, or in some cases solid minerals (geological sequestration), may also enhance vegetation productivity and subsequently soil carbon storage	[1••, 68, 69, 73••, 54]
Ocean alkalinization	Increase the alkalinity of the upper ocean to chemically increase the carbon storage capacity of seawater and thus, also increase CO_2_ uptake	[37•, 69, 72, 73••, 74–84]
Ocean fertilization	Add micronutrients like iron or macronutrients like nitrogen and phosphorus to increase phytoplankton growth (CO_2_ fixation) and ocean carbon storage via the biological pump (the transport of this fixed carbon into the deep ocean)	[37•, 85–90]
Artificial ocean upwelling	Use pipes or other methods to pump nutrient rich deep ocean water to the surface where it has a fertilizing effect; see ocean fertilization above	[37•, 40, 41, 91]

*Bioenergy and biochar can also be created from marine micro- or macro-algal biomass. However, no literature exists on how the C cycle would respond to large-scale (e.g., > 1Pg C) marine biomass growth and harvesting for these purposes

A further distinction can be made between CDR methods that seek to accelerate the uptake of atmospheric CO_2_ by enhancing natural sinks and methods that seek to engineer the removal and subsequent storage of CO_2_ [92, 93]. Examples of the former include increasing carbon storage in biomass through expanding forest cover (afforestation) [36, 37•], and accelerating the rate of natural weathering of rocks, which chemically removes CO_2_ from the atmosphere, by increasing their surface area (enhanced weathering) [1••, 68]. Examples of the latter include combining bioenergy with carbon capture and storage (BECCS) [1••, 94] and direct air capture (DAC) with storage [11]. BECCS uses biomass resources (e.g., energy crops or forestry residues) for energy conversion processes (e.g., combustion or gasification) and captures the CO_2_ during these processes. DAC uses machines to separate CO_2_ from the air. For both methods, the captured CO_2_ can be geologically stored or used as a chemical feedstock for the manufacture of long-lived products.

At present, CDR methods and technology are immature and untested at scale [95], so our understanding of their potential and impacts, including possible carbon cycle feedbacks, are reviewed from limited investigations. For example, many studies extrapolate from natural analogues or local applications, modeling and/or laboratory investigations, and in some cases a small number of pilot projects. However, these are insufficient for a full assessment of CDR and so there is a pressing need to advance research and, where relevant, the development of CDR technologies [96].

Fuss et al. [97] highlight several important CDR research priorities: (i) improving estimates of sustainable potentials, particularly for methods that require using large land areas or other limited resources; (ii) assessing the benefits and risks of different CDR methods to contribute towards climate stabilization; (iii) developing a governance model for CDR; and (iv) understanding the carbon cycle responses to CDR.

This article focuses on this last priority (iv) *understanding the carbon cycle responses to CDR*, specifically reviewing the latest research findings relating to CDR and the carbon cycle. The structure of this paper is as follows. We first describe the factors that control the response of the carbon cycle to perturbations; then collate and consider research findings into the overall effect of CDR on the carbon cycle with a description of the response to atmospheric CO_2_ removal; and finally examine the possible carbon cycle impacts of specific CDR methods with a focus on widely discussed and researched methods (Table 1). Other CDR methods, which have received less study (Table 2), are also briefly addressed. We conclude with a discussion of present unknowns and future research priorities.

**Table 2:**
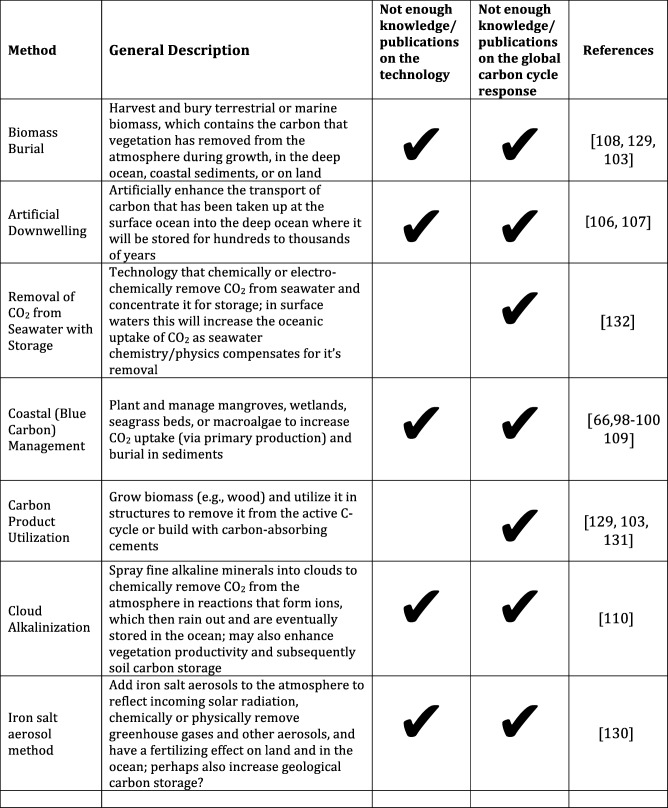
Proposed carbon dioxide removal methods for which there is insufficient literature to assess their response (< 2 publications) and confidently report on either the functional feasibility of the method (i.e., would it potentially work from an Earth system perspective) or on how the global carbon cycle would respond to the method.

## Factors Controlling the Carbon Cycle Response to Perturbations

The atmosphere, ocean, terrestrial biosphere, and geosphere hold a vast amount of carbon that is naturally exchanged between reservoirs (Fig. 1a). The exchanges of carbon between these reservoirs are mediated through biological, chemical, and physical mechanisms. Understanding how the carbon cycle has responded to past changes is key to understanding how it may respond to the ongoing anthropogenic perturbations. Here, we briefly describe the most relevant factors that control the carbon cycle response to anthropogenic perturbations. We focus only on those carbon reservoirs and processes that respond on timescales of up to a few centuries, thereby neglecting exchanges with the geological reservoir, except in cases of direct anthropogenic disturbance, e.g., fossil fuel use and engineered geological or geochemical storage of CO_2_.

## The Factors that Control the Response of the Ocean to Carbon Cycle Perturbations

The exchange of atmospheric CO_2_ with the ocean is through air-sea CO_2_ fluxes driven by the differences in the partial pressures (pCO_2_) between the atmosphere and surface ocean. The rate of the exchange depends on wind speed, temperature, and other factors, whereas the pCO_2_ gradient (if present) sets the magnitude and the direction of the carbon flux. In the case that a perturbation acts to reduce oceanic pCO_2_ to be lower than the atmosphere pCO_2_, e.g., when CO_2_ is added to the atmosphere via fossil fuel combustion, oceanic carbon uptake is enhanced. Conversely, if atmospheric pCO_2_ is reduced and is lower than that of the ocean, e.g., via DAC, this will lead to a transfer of carbon from the ocean into the atmosphere. As these CO_2_ fluxes are driven by pCO_2_ differences across the marine boundary layer, it is important to understand the processes that control how carbon is (re)distributed in the ocean, i.e., the factors that determine whether carbon stays near the surface or is sequestered into the deep ocean where it cannot readily be exchanged with the atmosphere.

The surface ocean pCO_2_ is controlled through the physical and biological carbon pumps [111]. The biological carbon pump describes the photosynthetic uptake of carbon at the surface, i.e., conversion from inorganic to organic carbon, and its subsequent transport (in particular via sinking particles) to the deep ocean. This sequestration of photosynthetically fixed carbon is inefficient with only a small proportion (10–15%) making it into the deep ocean (below the surface mixed layer that is in contact with the atmosphere) on annual timescales. The remaining 85–90% of the organic carbon is remineralized (i.e., broken down and transformed into an inorganic form via respiration). Photosynthesis is sensitive to changes in temperature, nutrients, and light availability. The rate of remineralization is sensitive to changes in temperature [112], the available quantity and quality of organic matter, and other factors such as oxygen levels and, for calcium-carbonate containing matter, seawater chemistry.

The physical carbon pump describes the physico-chemical processes that move dissolved inorganic carbon from the surface to the interior ocean. The magnitude of this pump is determined by CO_2_ solubility, which depends on temperature (i.e., the warmer the ocean the less soluble CO_2_ is in seawater) and carbonate chemistry, as well as physical ocean circulation, which transports the dissolved inorganic carbon. Ocean circulation can change in response to changes in wind stress, which particularly affects the upper ocean, and changes in buoyancy fluxes in response to warming and alterations in the hydrological cycle such as melting of ice, which can impact the formation of deep waters and the deep overturning circulation.

Although the general large-scale mechanisms of marine carbon cycling are well established, there remain processes that are less well understood and poorly quantified [111]. These lead to uncertainties in the present-day ocean CO_2_ uptake (3 ± 0.5 Gt C year^−1^) [113], and in predicting the future evolution of the marine carbon cycle in response to climate and environmental change.

## The Factors that Control the Response of the Land to Carbon Cycle Perturbations

Biological processes primarily control the exchange of atmospheric CO_2_ with the land, where the majority of the carbon is stored in soils and permafrost [3]. CO_2_ is removed from the atmosphere by plant photosynthesis and primarily returned to the atmosphere by respiration and other processes such as fire. As long as primary production (GPP; i.e., gross photosynthetic carbon fixation) is greater than carbon losses due to respiration (autotrophic and heterotrophic) and processes such as fire, the land will be a carbon sink as it is today [113]. However, if a climate-carbon cycle perturbation such as an ocean-based CDR reduces GPP and increases or maintains rates of carbon losses that are higher than GPP, then the land could become a source of CO_2_. The factors that control the balance between terrestrial carbon uptake and loss are described below.

Photosynthesis rates are sensitive to changes in temperature and the availability of nutrients, light, and water. If nutrients, light, and water are available, the photosynthetic rate will increase with temperature until a maximum species-specific rate is reached, after which the photosynthetic rate decreases. Plant photosynthesis is also directly affected by the concentration of CO_2_ in the atmosphere through the CO_2_ fertilization effect. Higher concentrations of CO_2_ allow more photosynthesis and reduced canopy transpiration, which in turn lead to increased plant water use efficiency and reduced fluxes of surface latent heat [3]. This climate-carbon cycle feedback (i.e., CO_2_ fertilization) is thought to play a major role in determining how the land carbon sink responds to changes in atmospheric CO_2_ [114]. Changes in the amount, density, or type of vegetation (e.g., by deforestation) will also alter photosynthetic carbon uptake. In addition, these changes may have physical climate feedbacks (e.g., through albedo changes that can cause warming or cooling), thereby having a further impact upon vegetation [115]. Past land use and land cover changes have already had a large effect on the climate and carbon cycle and will play a role in determining how the land responds to any future perturbations [116–118].

While the rate of autotrophic (plant) respiration is sensitive to the factors that control photosynthesis, heterotrophic (animal, fungi, protist, and non-photosynthetic bacteria) respiration is sensitive to changes in temperature, moisture, and the available quantity and quality of organic matter and nutrients [53, 119, 120]. Changes in vegetation may alter the organic matter available for heterotrophic consumption, thereby impacting respiration rates. Soil disturbance (e.g., from land use change and fire) can have a range of impacts on soil organic matter and can impact the net flux of carbon to the atmosphere.

The terrestrial carbon cycle and present-day CO_2_ sink is less well constrained than that of the ocean with an estimated atmosphere to land flux of 1.9 ± 0.9 Gt C year^−1^ [113]. How the terrestrial carbon cycle will be impacted in the future in response to climate and land management changes, including the possible application of land-based CDR, is even more uncertain [121]. For example, in high CO_2_ emission scenarios, some models suggest that the land will become a source of CO_2_ while in others it remains a sink [122]. Major uncertainties remain in the projected changes in the availability of water and nutrients, but also in the physiological and, eventually, adaptive, and evolutionary responses of plants to enhanced atmospheric CO_2_ and temperatures, as well as other environmental changes.

Carbon stored in permafrost is also sensitive to changes in the climate. If permafrost thaws, then it can be released/transformed into CO_2_ and other greenhouse gases (in particular methane), though details in seasonal temperatures as well as the hydrological cycle may be crucial to determining the exact fate of the organic matter. At present, many aspects of these processes remain poorly understood [123].

Freshwater bodies also exchange carbon with the atmosphere across the air-water interface, with these exchanges driven by biological, chemical, and physical processes that are sensitive to many of the factors described above (e.g., temperature, light, hydrology, and nutrient and organic matter input). A significant amount of terrestrial carbon is also transported through freshwater bodies and eventually reaches the ocean if not remineralized or buried along the way [12, 124].

## The Response of the Carbon Cycle to Anthropogenic Perturbations

### Industrial Era Perturbations

The carbon cycle has responded to industrial era anthropogenic perturbations by redistributing carbon within and across reservoirs (Fig. 1b) in response to land use changes, other environmental disturbances (e.g., fires, nutrient additions, invasive species), and CO_2_ emissions. Only around 42% of the CO_2_ added to the atmosphere since the industrial revolution has remained in the atmosphere (termed the airborne fraction), with the remainder approximately evenly taken up by the land and ocean, respectively [113]. Carbon fluxes from the land to ocean have also increased [12].

### Atmospheric Carbon Dioxide Removal Perturbations

In this section, we review what has been learned about the response of the carbon cycle to CDR from idealized model simulations, which in most cases are analogous to technology that directly removes CO_2_ from the atmosphere [11] and permanently stores it elsewhere (DAC; Table 1). Note that in the literature, CDR is often defined as the *gross* removal of carbon (e.g., 1 GtCO_2_ removed/sequestered from the atmosphere and stored in geological reservoirs) rather than the *net* removal of carbon from the atmosphere (e.g., the net impact is much less when the response of the carbon reservoirs is accounted for). While understanding the gross sequestration potential of a method is important, understanding the net removal of CO_2_ from the atmosphere is the key to evaluating the efficacy (defined here as a change in atmospheric CO_2_ per unit CDR) of that method as a climate change mitigation measure.

The earliest idealized simulations of CDR, in models with a carbon cycle, prescribed either massive instantaneous CO_2_ removals (negative pulses) or large decreases (e.g., 1% year^−1^) in atmospheric CO_2_ [13, 14]. Following this, studies were conducted with more complex pathways of atmospheric CO_2_ reduction [15••, 16, 17•, 18••] and additional components such as permafrost [19]. Some studies also focused on the response of a particular carbon reservoir such as the ocean [20, 21]. As a result of these studies, we have a growing understanding of how the carbon cycle responds to atmospheric CDR. Models project that when net CO_2_ emissions under CDR are positive, but start to decline, the land and ocean carbon sinks will begin to weaken and take up less CO_2_ (Fig. 1c). Note that these responses may not be driven entirely by CO_2_ forcing as other factors such as a changing climate also affect the strength of these sinks. At some point, as net CO_2_ emissions decline, carbon sink uptake will exceed emissions input and the atmospheric CO_2_ concentration will begin to decline. Atmospheric CO_2_ will continue to decline once atmospheric CDR is deployed at a large enough scale that net CO_2_ emissions become negative. At some point (years to centuries; and depending on the CDR rates), the oceans and land may become net emitters of CO_2_ back to the atmosphere (Fig. 1c). Note that the land response is uncertain with some models suggesting that the land may never become a source of CO_2_. This uncertainty is not surprising given that there is also a large spread in the simulated terrestrial carbon cycle response to increasing atmospheric CO_2_ [122] because of differences in model representations of system processes [125]. Also uncertain are the timescales of the response, as changing land and ocean sinks to sources does not occur instantaneously (e.g., transient climate change simulations have shown that the sinks may continue to respond to the prior emission trajectory for years to centuries before responding to CDR [15••, 18••]). More specifically, land responses may take years to decades if only some land components, e.g., vegetation, have been perturbed and potentially longer (centuries to millennia) if other components like the permafrost carbon pool have been perturbed. The ocean also generally takes a long time (decades to millennia) to respond. For all carbon reservoirs, the response depends on the rate and/or amount of CDR and the prior state of the climate and carbon cycle [17•, 18••].

This carbon cycle response tends to oppose CDR [15••, 18••]. For example, as illustrated in Fig. 4 of Keller et al. [126], instantly removing 100 Gt CO_2_ from the atmosphere in Earth system models at a pre-industrial steady-state will only reduce the atmospheric CO_2_ concentration by 100 Gt CO_2_ immediately following the removal. After 100 years, atmospheric CO_2_ is only ~25 Gt CO_2_ lower because carbon is gradually released by the ocean and land in opposition to atmospheric CDR.

It is conceivable that CDR will be discontinued at some point, e.g., after atmospheric CO_2_ has reached a desired level. The carbon cycle response to cessation will depend on the state of the climate and the cumulative amount of CDR. In overshoot emission scenarios simulations, models show that when CDR ceases and CO_2_ emissions are zero, the ocean is likely to again become a carbon sink (if it had stopped being so in the first place) as the oceanic carbon pumps drive net oceanic pCO_2_ to again become lower than the atmosphere pCO_2_. Because of slow ocean circulation, it will take several millennia before the deep ocean carbon cycle recovers from past and any future anthropogenic perturbations [21]. The response of the land is less clear, with some simulations showing that it may continue to lose carbon to the atmosphere, albeit at a lower rate, or that it may again revert to a carbon sink [15••, 17•, 18••]. These responses may take years to decades on land, unless soil carbon pools have been perturbed in which case it may take centuries to millennia for any change of sign to occur.

Recent studies find that the efficacy of CDR depends on the scenario of removal and the state of the Earth system [15••, 17•, 18••]. When the background climate and carbon cycle state is the same (i.e., in equilibrium/at steady-state), more CDR results in stronger opposition (outgassing) from the ocean and land. CDR efficacy also changes over time in conjunction with co-occurring climate-carbon cycle feedbacks. If the background climate and carbon cycle state has been perturbed (i.e., is out of equilibrium), then the efficacy of CDR depends on the magnitude of perturbation (i.e., CDR efficacy is state dependent and the same level of CDR may be more or less effective in different scenarios). As described in Jones et al. [15••], a “perturbation airborne fraction” metric can be calculated to estimate the effectiveness of CDR for different background climate and carbon cycle states. In either case (steady-state vs. a perturbed state), there are likely to be large regional differences within reservoirs due to the CDR carbon cycle responses, e.g., in terrestrial photosynthesis and respiration or oceanic air-sea gas exchange [18••, 20].

However, in the longer term (centuries to millennia), the carbon cycle response to the same cumulative amount of CDR (if permanently removed) appears to be pathway independent (if the background climate and carbon cycle state are the same), and determined only by cumulative CO_2_ emissions [18••]. However, this does not account for the possibility of there being un-represented thresholds or tipping elements in the climate system [38].

## CDR Method-Specific Carbon Cycle Responses

**Fig. 2:**
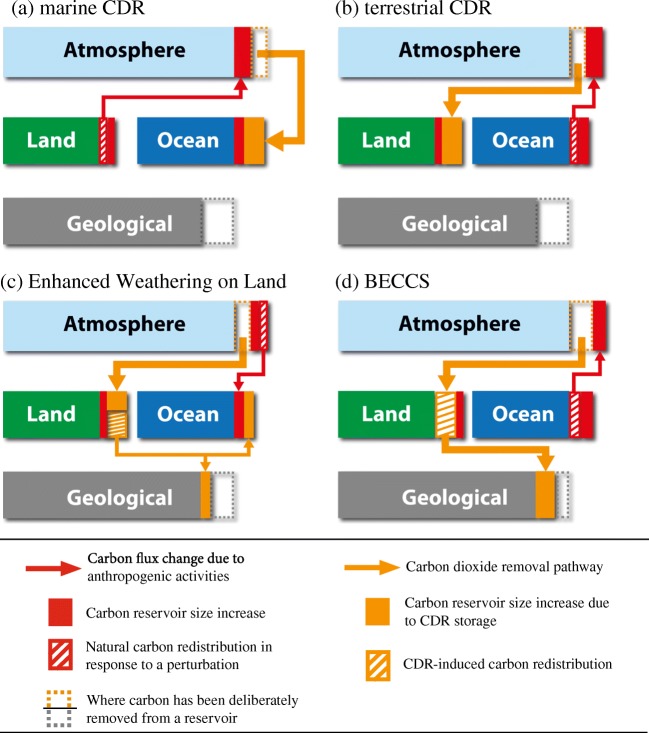
Schematic representation of how carbon flows among atmospheric, land, ocean, and geological reservoirs may change due to prolonged “net negative emission” carbon dioxide removal (CDR) from (**a**) marine sink enhancement methods such as ocean fertilization or alkalinity enhancement; (**b**) terrestrial sink enhancement methods such as afforestation/reforestation, biochar, or soil carbon management; (**c**) enhanced weathering on land; and (**d**) bioenergy with carbon capture and storage (BECCS). All carbon exchanges depicted in Fig. 1a also occur here. Note that when net emissions are negative, it is still possible to have some fossil fuel emissions, but these are not depicted here. Adapted from [1••, 71•]

Methods proposed to enhance ocean or land carbon sinks aim to increase the flux of carbon from the atmosphere into that reservoir (Tables 1 and 2). More technical CDR methods, including those that combine natural processes and technology, are designed to directly remove carbon from a reservoir and isolate it from the climate system, e.g., geological reservoir storage [2]. For almost all evaluated methods, this results in reduced carbon uptake or even carbon loss from another reservoir (Figs. 1 and 2). All methods will also either directly or indirectly redistribute carbon within reservoirs. For many methods, storage is an issue as sequestration sites, e.g., soils, may have limited capacity or only temporarily or inefficiently hold the carbon, i.e., the issue of permanence [2, 71•].

### Ocean-Based CDR

Artificial ocean alkalinization (AOA), a proposed CDR method that aims to reduce surface seawater pCO_2_ and thereby enhance oceanic carbon uptake (Table 1), has recently been reviewed in several publications [69, 72, 73••]. Model simulations of AOA [37•, 39, 74–81] have found AOA to have, theoretically, a high carbon sequestration potential, and also explored how it might affect ocean carbonate chemistry. However, for the most widely proposed practical application of AOA using crushed olivine minerals, the chemistry is more complex than the simplified addition of alkalinity simulated by most models [82, 83]. Only a few studies have investigated the additional fertilizing effect of nutrient additions from olivine-based AOA [75, 76]. Albright et al. [84] recently explored AOA in a field setting at very small scale by adding alkalinity to a reef and found that it is possible to locally return seawater chemistry to a near pre-industrial state. Studies with Earth system models have all shown that in high CO_2_ emission scenarios, the terrestrial biosphere loses carbon, via climate-carbon cycle feedbacks, as atmospheric CO_2_ and temperatures decrease in response to increasing oceanic CO_2_ uptake [37•, 39] (Fig. 2a). Furthermore, the mean state of the ocean carbonate chemistry determines the magnitude of the Earth system response to AOA [80]. There appears to be little cessation effect as CO_2_ taken up by the ocean as a result of AOA remains in the ocean upon AOA termination [37•, 72, 77], although any olivine-related fertilizing effects are temporary [75].

Enhancement of the biological pump as a means of atmospheric CDR has centered on macro- and micro-nutrient ocean fertilization (Table 1). Keller et al. [37•] found that as in many earlier studies, there is limited potential for iron fertilization to draw down CO_2_. Moreover, it has recently been observed in regions of natural iron fertilization that the carbonate counter pump (i.e., the biological precipitation of the carbonate shells of some species, followed by sinking of particulate inorganic carbon, which increases surface ocean pCO_2_ on centennial to millennial timescales) may reduce iron fertilization-induced C sequestration by 6–32% [85]. Oschlies et al. [86] and Keller et al. [37•] show that when iron is added during a high CO_2_ emission scenario, fertilization-induced atmospheric CO_2_ drawdown is initially opposed by a net loss of carbon from the terrestrial biosphere (Fig. 2a), via climate-carbon cycle feedbacks, as atmospheric CO_2_ and temperatures decrease. This phenomenon lasts until it is countered by a decreasing sequestration efficacy (i.e., fertilization becomes less effective at enhancing oceanic carbon uptake) and increased terrestrial uptake as a result of CO_2_ fertilization. They also show that in the fertilized region there is a decline in pH and an increase in pCO_2_. Carbon cycling is also affected in other regions of the ocean because of reduced nutrient availability following enhanced biological production. Furthermore, if ocean iron fertilization is terminated, carbon may remain in the ocean for a few decades [37•], but after 100 years up to a third of it could be potentially returned the atmosphere [87].

Other studies have estimated the carbon sequestration potential of nitrogen and phosphorus fertilization [88–90]. While these macro-nutrient fertilization studies explore some of the factors that would determine carbon sequestration and estimate ocean carbon uptake, they do not fully explore the response of the global carbon cycle.

Artificial ocean upwelling (Table 1) as a biological pump enhancement has been investigated by Oschlies et al. [91] and reviewed by Bauman et al. [40] and Pan et al. [41]. Many of the impacts are the same as for ocean fertilization (see above) since it is the goal of this method to fertilize upper ocean biology with upwelled nutrients. However, upwelling also directly affects the carbon cycle since in most places the deep water that is pumped up contains more CO_2_ than the surface ocean, part of which may outgas into the atmosphere if the additional C is not consumed by phytoplankton. Deeper waters are also cooler than the surface ocean, and when pumped up have a cooling effect. In model simulations, this has been shown to increase terrestrial carbon uptake via reduced heterotrophic respiration at lower temperatures, i.e., there is no terrestrial loss of C as with other ocean-based CDR methods depicted in Fig 2a [37•]. Indeed, in model studies, artificial upwelling sequesters most (80% on centennial timescales) of the carbon drawn down from the atmosphere on land [88], and may therefore not be viewed primarily as ocean method. When artificial upwelling is stopped, the sequestered carbon is returned to the atmosphere within a few decades, and re-adjustment of the planetary energy budget leads to global mean temperatures even higher than in a world that had never experienced artificial upwelling [91].

### Terrestrial CDR

Although there are many studies on afforestation/reforestation (Table 1), very few of them quantify how the global carbon cycle responds from a CDR perspective. Most studies have instead focused either on carbon sequestration potential [42–44] and/or on the climatic trade-off between terrestrial CO_2_ uptake and biogeophysical change (e.g., in albedo) [1••, 37•, 45–50]. This trade-off determines how effective afforestation/reforestation is at cooling the climate and so impacts temperature-sensitive carbon cycle processes. Sonntag et al. [36, 79] recently showed that during simulated reforestation climate-carbon cycle feedbacks, such as CO_2_ fertilization, only slightly offset carbon sequestration and reductions in atmospheric CO_2_. Studies [37•, 51] have also investigated how atmospheric circulation and the water cycle would change with irrigated afforestation, which has carbon cycle implications. To date, the evidence indicates that strengthening the terrestrial carbon sink via afforestation/reforestation will weaken the ocean sink or even make it a CO_2_ source as atmospheric CO_2_ decreases (Fig. 2b) [22, 37•, 52, 60, 70]. Afforestation/reforestation also has limited carbon storage capacity and even after saturation (i.e., when the forest reaches a state of equilibrium and carbon uptake is balanced by loss), management must continue to prevent disturbances from releasing sequestered carbon [71•].

Much of what is known about how enhanced terrestrial weathering (Table 1) would affect the global carbon cycle is derived from our understanding of natural chemical weathering [54, 69, 73••] as well as recent modeling studies [68]. Weathering alters soil chemistry (e.g., increasing pH), which may increase nutrient availability, and can potentially add toxic trace metals to the environment. Soil composition may also be altered by the formation of minerals. These effects can impact vegetation, often positively (i.e., enhancing growth), and lead to changes in soil hydrology and carbon cycling. Nearby water bodies will be affected as well when weathering products are transported into them. Carbon sequestration estimates are poorly constrained, although likely low [1••] and with the exception of Taylor et al. [68] most studies do not account for global carbon cycle responses. To date, our understanding of the carbon cycle response to enhanced terrestrial weathering is that it removes CO_2_ from the atmosphere and redistributes it to the land, ocean, and geological reservoirs (Fig 2c). This redistribution may initially cause the ocean carbon sink to weaken or even to change from a sink to a source of CO_2_ as atmospheric CO_2_ decreases [68]. However, if enough weathering products are transported to the ocean such that alkalinity increases, this will eventually enhance ocean carbon uptake (see section on AOA). Most sequestered carbon will ultimately be permanently stored in the ocean or geological formations and the method is not subject to reversal upon cessation [68, 73••].

The effects of biochar or other measures to manage soil carbon (Table 1) on the global carbon cycle are poorly known, with almost all studies limited to impacts on the terrestrial carbon cycle and GHG exchange with the atmosphere [61, 62]. These measures, which are too numerous to review individually here, affect the terrestrial carbon cycle and atmosphere mainly through biophysical (e.g., albedo, hydrological) and biogeochemical (e.g., nutrients, the release of other GHGs) processes [23, 61–65, 71•, 120]. Although biochar can also be made from marine biomass [55, 56, 66], no studies have investigated how transferring large amounts of carbon from the ocean to land would impact carbon cycling. There have been many estimates of the carbon sequestration potential of these methods [24, 25, 57–59, 62, 65, 67, 71•], with most suggesting a low total potential. CDR through soil carbon management, and to a lesser extent biochar, is limited by terrestrial carbon storage capacity and even after saturation (i.e., when the system reaches a state of equilibrium and carbon uptake is balanced by loss) some management must continue or leakage could occur [71•]. To date, our understanding of the global carbon cycle response to biochar or soil carbon management suggests that strengthening the terrestrial carbon sink should weaken the ocean carbon sink or lead to outgassing as atmospheric CO_2_ decreases (Fig. 2b).

BECCS (Table 1) is the CDR method most often assumed in climate change mitigation scenarios [26] and there are many recent carbon sequestration potential estimates [1••, 27–30, 60, 65]. However, as far as we are aware, global carbon cycle responses to BECCS have only been fully quantified by Muri [31], although there have been offline modeling efforts [60]. Research instead usually focuses either on biomass plantations (simulated with offline terrestrial models [29, 30, 32–34, 60]) or the ESM response to atmospheric CO_2_ removal, where BECCS is treated as DAC, e.g., Jones et al. [15••]. In these DAC-like studies, biomass plantations may be prescribed, but the harvest products are returned to the terrestrial carbon pool as litter or crop residues, rather than being removed from the simulated carbon cycle. Consequently, these studies likely underestimate soil carbon losses resulting from bioenergy cropland expansion. The impacts of bioenergy plantations on soil carbon stocks are likely to be mixed and depend heavily on previous land cover [35]. Increases in soil carbon can remove CO_2_ from the atmosphere, representing a carbon sink regardless of whether CCS is performed on the harvested biomass, e.g., as shown in simulations where biomass is grown to substitute bioenergy for fossil fuels [127]. However, carbon stored in soils is vulnerable to future changes in land use and does not represent the same permanence as geological CO_2_ storage. All terrestrial biomass plantation studies have shown that there would be biogeophysical (e.g., albedo, hydrological) and biogeochemical (e.g., nutrients) changes that impacts the carbon cycle [1••, 28–30, 33, 34, 65]. From a global carbon cycle perspective, the net result of terrestrial-based BECCS is to move carbon from the atmosphere to the terrestrial biosphere and then to permanent geological storage (Fig. 2d). This will weaken the ocean carbon sink or even result in it switching to a CO_2_ source as atmospheric CO_2_ decreases. Note that there have also been proposals to utilize marine biomass (macroalgae or microalgae) as the BECCS fuel feedstock [101, 102, 128]. This would have different impacts on the carbon cycle than the use of a terrestrial feedstock, but these have not yet been quantified.

### CDR Carbon Storage and the Carbon Cycle

Ideally, any captured carbon will be permanently stored (e.g., in geological reservoirs), and this underpins all technical methods, e.g., BECCS and DAC (Fig. 2c, d). Permanent storage capacity for sequestered CO_2_ appears adequate to technically match current fossil fuel reserves (i.e., the amount that could be emitted and countered by CDR) [2]. However, it is not clear whether storage can be accessed fast enough or is appropriately located to meet the mitigation demands for fossil fuels with carbon capture and storage and CDR, so the use of more temporary storage sites has also been investigated. Reith et al. [104] recently revisited the idea of using the deep ocean as a site to store carbon from DAC and quantified leakage rates and terrestrial and ocean carbon cycle responses. They found that their targeted atmospheric CO_2_ reductions, which were equivalent to the amounts of CO_2_ injected, were 16–30% lower on decadal to centennial timescales because of leakage and land and ocean carbon back fluxes that occurred in response to lowering atmospheric CO_2_. Responses like this need to be taken into account when considering any storage sites that are not permanent.

### Solar Radiation Management Effects on the Carbon Cycle

Solar radiation management (SRM), a term that describes methods that attempt to reduce climate warming by increasing the reflection of incoming short-wave solar radiation back into space, is not intended to reduce CO_2_. However, it may do so by impacting the carbon cycle via reduced surface temperatures, altered diffusive radiation, or by limiting carbon cycle feedbacks that amplify climate responses to emissions (i.e., feedbacks that increase atmospheric CO_2_). These effects are not explored here, but reviewed by Cao [105].

## Knowledge Gaps

There are many carbon cycle uncertainties for all the CDR methods discussed above. Uncertainties may be highest for methods that have been investigated in only a few studies, or are merely conceptual proposals (Table 2). Such methods include artificial downwelling [106, 107], coastal (blue carbon) sink management [66, 98–100, 109], cloud alkalinization [110], burial of terrestrial biomass on land or in the deep ocean [103, 108, 129], iron salt aerosol additions [130], storing carbon in structural materials [103, 131], (e.g., by building with wood or carbon absorbing cements or utilizing it in other products), or extracting CO_2_ directly from seawater [132]. There is also little knowledge about the effects that may emerge when any of these methods is scaled up to have globally significant impact. These include not only biogeochemical but also ecological as well as economical feedbacks. Biological methods will directly have to account for not only possible impacts on ecology, but also on possible effects of CDR on climate trajectories and therefore the different capacity of species to adapt and evolve.

## Summary

When carbon is deliberately transferred to or removed from a reservoir, the carbon cycle responds by redistributing the carbon in other reservoirs (Figs. 1 and 2), as well as within them, as biological, chemical, and physical processes adjust to the changing quantities of carbon within the reservoir and steeper gradients at the reservoir interfaces. By reducing the greenhouse effect, which leads to cooling, CDR also triggers climate-carbon cycle feedbacks. Together, these responses affect the efficacy of CDR and mean that removing 1 Gt of CO_2_ from the atmosphere will not ultimately reduce the atmospheric CO_2_ concentration by 1 Gt. The short-term efficacy of any CDR method depends on the state of the climate system and the carbon cycle. For many methods, environmental factors (e.g., light or nutrient availability) are also important. However, in the long run (multi-millennial) for methods that can permanently sequester carbon, it appears that the carbon cycle response to the same cumulative amounts of CDR is, to first order, pathway independent (if the background climate and carbon cycle state is the same) and determined only by cumulative CO_2_ emissions. Although, this does not account for the possibility of there being thresholds or tipping elements in the climate system [38].

In addition to the general response of the carbon cycle to CDR (i.e., reservoir scale redistribution), there are a wide variety of CDR method-specific carbon cycle impacts. Many of these significantly alter the carbon cycle at the site of deployment, but they can also have large regional or even global-scale impacts. Storage is an important issue for most methods as sequestration sites might only temporarily or inefficiently hold the carbon and/or have limited capacity [2].

Although CDR has been simulated in idealized scenarios that remove massive amounts of carbon in a short time, studies that apply more realistic constraints show that most sequestration rates are likely to be low and limited by many factors, such as land area or local environmental conditions (e.g., temperature, hydrology, chemistry) that impact reaction or growth rates [1••, 28, 29, 36, 37•, 68, 71•, 73••]. This does not mean that CDR could not potentially work, just that most methods are relatively slow acting and might take many decades to centuries to reduce atmospheric CO_2_ to some desired level. The few methods that have the theoretical potential to rapidly remove more CO_2_ remain technologically immature and would likely take decades to be fully developed and deployed at climatically relevant scales.

## Conclusion

CDR is increasingly and widely applied in global and regional decarbonization scenarios used to inform policy and economic discussions on climate change mitigation. As a result, improved understanding of the response of the Earth system to CDR is needed to inform the research and development, possible policy frameworks, and the proper accounting of CDR.

For anthropogenic CO_2_ emissions, it is well established that only a fraction of the emissions effectively remains in the atmosphere because carbon cycle feedbacks adjust the carbon cycle to the anthropogenic perturbation by redistributing carbon among the interacting oceanic, terrestrial, and atmosphere reservoirs. Because of the different response times of terrestrial and oceanic carbon reservoirs, this partitioning changes with time and also depends on the state of the climate system. The same processes apply when CO_2_ is removed from the atmosphere, yielding an airborne fraction of CO_2_ removal, i.e., the perturbed airborne fraction [15••]. As a corollary, taking CO_2_ out of the atmosphere will lead to backfluxes of CO_2_ from the ocean and the land. The services that the land and ocean have provided in taking up a substantial portion of anthropogenic CO_2_ emissions can be viewed as a “carbon debt” that will have to be started to be paid back if large-scale CDR is ever deployed.

The carbon cycle response to CDR and its impact on CDR efficacy will also have to be considered in the comparative assessment of different methods. Two options for estimating efficacy appear straightforward: measuring CDR in terms of (i) carbon stored (e.g., in biomass or chemically inert forms of carbon on land or in the ocean) or (ii) net changes in atmospheric CO_2_. The first option (i) is equivalent to measuring the local fluxes of CO_2_ from the atmosphere, and thus corresponds to the way anthropogenic CO_2_ emissions are measured. For monitoring and verification of CDR, accounting based on local measurements would, however, pose substantial challenges, especially if future leakage (lack of permanence) is possible. The latter option (ii) can more easily be quantified. However, attributing net changes in atmospheric CO_2_ to any particular CDR method, especially if multiple methods were deployed, would be problematic. It is likely that aggregated methods will be required to verify and monitor any CDR deployment that, according to current understanding, will be required to meet internationally agreed climate goals. The carbon cycle responses discussed in this review will have to be carefully considered in assessments of CDR options and, if deployed, in the accounting process.

Our literature review shows that relatively few studies have quantified how the global carbon cycle responds to CDR. Far more studies quantify gross carbon sequestration than net atmospheric CO_2_ reduction that accounts for the response of other reservoirs. This is likely because global carbon cycle responses can only be evaluated with Earth system models that include these reservoirs, while gross carbon sequestration and local carbon cycle impacts can often be quantified with laboratory or field experiments and simpler models that resolve only parts of the full Earth system. Nonetheless, net CDR efficacy must be properly evaluated as it lies at the heart of the climate mitigation potential of CDR.

This review has made it clear that further and targeted research is urgently needed to better constrain the carbon cycle response to CDR. This research should focus on: (i) understanding the underlying mechanisms involved in each CDR method (i.e., improving the quantification of relevant biological, chemical, and physical processes); (ii) understanding and reducing the large model spread that occurs when the carbon cycle response to perturbations is simulated (e.g., reducing the uncertainty in the sensitivity of carbon fluxes and storage to environmental conditions); (iii) quantifying how reversible the climate and carbon cycle are with respect to perturbations by CO_2_ emissions and CDR; and (iv) developing a methodology to evaluate and monitor CDR and its impacts on the carbon cycle and climate system, in order to allow for reliable accounting of CDR contributions to managing atmospheric CO_2_ concentrations.

For (i) some understanding can be gained through more detailed simulations, however, in many cases laboratory, mesocosm, or field experiments will be needed to make advances. In combination with model assessment and intercomparison studies (such as the carbon dioxide removal model intercomparison project, CDRMIP; [126]), this will help to reduce uncertainties in simulating CDR (e.g., by providing better parameterizations and by developing more realistic models). Model intercomparison and perturbed parameter ensemble studies will also provide information to address (ii) and eventually our representation of CDR in Earth system simulations, thereby providing increasing confidence in evaluating reversibility (iii) using models. Such advances require well-funded and sustained international research programs.

Given the potential large impacts (side effects), costs, and deployment requirements (e.g., land area) of many methods, an interdisciplinary approach not limited to social sciences, international law, and environmental ethics is required to develop the relevant assessment methods and tools needed for (iv). Some of this research overlaps with what is already being done to address the World Climate Research Program Grand Science Challenges; however, many aspects of CDR research are unique and will require building new interdisciplinary research consortia and dedicated research programs.
